# Difficulties in assessing the medical fitness of workers with mood disorders : A study of 101 cases

**DOI:** 10.1192/j.eurpsy.2024.892

**Published:** 2024-08-27

**Authors:** S. Chemingui, D. Brahim, M. Mersni, N. Mechergui, M. Methni, H. Ben Said, I. Youssef, G. Bahri, I. Yaich, C. Ben Said, N. Bram, N. Ladhari

**Affiliations:** ^1^Occupational pathology and fitness for work department, Charles Nicolle Hospital; ^2^Occupational pathology and fitness for work department Charles Nicolle Hospital, tunis, Tunisia; ^3^Forensic Psychiatry department, Razi Hospital, La Manouba, Tunis, Tunisia

## Abstract

**Introduction:**

Assessing the medical fitness of workers with mood disorders remains a topical issue, because of its organizational, socioeconomic and professional impact.

**Objectives:**

To assess the medical and occupational characteristics of workers with mood disorders.

To evaluate the impact of these psychiatric disorders on the medical decision of fitness for work.

**Methods:**

Descriptive and retrospective study, over six years (January 1, 2018 to August 30, 2023) including all medical records of workers with mood disorders (bipolar disorder, anxiety disorder, and depression), referred to the occupational department of the Charles-Nicolle Hospital in Tunis for a medical fitness for work.

**Results:**

The study included 101 patients, mostly female (sex ratio = 0.4), with a mean age of 43.3 ± 9.2 years. The most represented sector of activity was health care. The participants were mainly nurses (25%), followed by technicians (22%) and workers (21%). The mean job seniority was 16.5 ± 9.3 years. A pathological history was found in 74.3% of cases, of which 47.5% were psychiatric disorders. Mood disorders identified in our population were: bipolar disorder (53.5%), anxiety disorder (43.5%), and depression (3%). After medical examination and the decision of treating physician, 39% of the patients (N=39) were declared fit for work, and 31.4% (N=32) were fit with ergonomic adjustments. These accomodations consisted mainly of night shift exemptions in 75% of cases. Temporary unfitness was declared in 24 patients (23.6%). Job mutation was recommended for four patients. Early retirement due to invalidity was proposed for two patients.

**Conclusions:**

The decision on the medical fitness of workers with psychiatric disorders remains a delicate issue that requires the attention of both legislators and occupational health practitioners.

**Disclosure of Interest:**

None Declared

